# Inhibitory receptor expression on memory CD8 T cells following Ad vector immunization

**DOI:** 10.1016/j.vaccine.2016.08.048

**Published:** 2016-09-22

**Authors:** Pablo Penaloza-MacMaster, Quazim A. Alayo, Joshua Ra, Nicholas M. Provine, Rafael Larocca, Benjamin Lee, Dan H. Barouch

**Affiliations:** a Center for Virology and Vaccine Research, Beth Israel Deaconess Medical Center, Boston, MA 02215, United States; b Ragon Institute of MGH, MIT, and Harvard, Boston, MA 02114, United States

**Keywords:** Adenovirus, Vector, PD-1, HIV-1

## Abstract

T cells are an important component of immune responses, and their function is influenced by their expression of inhibitory receptors. Immunization with alternative serotype adenovirus (Ad) vectors induces highly functional T cell responses with lower programmed cell death 1 (PD-1) expression and increased boostability relative to Ad5 vectors. However, a detailed phenotypic characterization of other inhibitory receptors is lacking, and it is unknown whether Ad5-induced CD8 T cells eventually recover function with time. In this report, we measure the expression of various inhibitory receptors and memory markers during early and late time points following vaccination with Ad5 and alternative serotype Ad vectors. CD8 T cells induced by Ad5 exhibited increased expression of the inhibitory receptor Tim-3 and showed decreased central memory differentiation as compared with alternative serotype Ad vectors, even a year following immunization. Moreover, relative to Ad5-primed mice, Ad26-primed mice exhibited substantially improved recall of SIV Gag-specific CD8 T cell responses following heterologous boosting with MVA or Ad35 vectors. We also demonstrate that low doses of Ad5 priming resulted in more boostable immune responses with lower PD-1 expression as compared to high Ad5 doses, suggesting a role for vector dose in influencing immune dysfunction following Ad5 vaccination. These data suggest that Ad5 vectors induce a long-term pattern of immune exhaustion that can be partly overcome by lowering vector dose and modulating inhibitory signals.

## 1 Introduction

Adenovirus (Ad) vectors are important vaccine platforms due to their productive capacity and immunogenicity [1], [2], [3], [4]. A disadvantage of Ad5 vectors is their high seroprevalence [1], [5], which led the development of Ad vectors from alternative serotypes [6]. We have shown that immunization with alternative serotype Ad vectors provides immune protection against stringent SIV challenges in rhesus monkeys [7], [8]. Moreover, immunization with Ad5, but not alternative serotype Ad vectors (such as Ad26, Ad35, and Ad48), results in upregulation of programmed cell death 1 (PD-1) on vaccine-elicited CD8 T cells and reduced recall responses [9]. We therefore examined the expression of other co-inhibitory receptors and homeostatic survival markers on CD8 T cell responses following vaccination with Ad5 or alternative serotype Ad vectors, including Ad26, which has shown promise in pre-clinical HIV vaccine studies and is being evaluated in clinical trials [10], [11], [12].

Expression of CD127 (the IL-7Rα chain) selectively marks effector CD8 T cells that survive the contraction phase and give rise to memory CD8 T cells [13]. Moreover, memory CD8 T cells that express CD62L (referred to as central memory CD8 T cells) exhibit greater anamnestic capacity and provide enhanced immune protection following various pathogen challenges [14]. CD8 T cell function is also dependent on the expression of inhibitory receptors [15]. PD-1 is a important inhibitory receptor that negatively regulates T cell activation and cytokine production [15], [16], [17], [18], [19], [20], [21], [22].

We have shown that alternative serotype Ad vectors induce memory T cells with enhanced functionality and reduced PD-1 expression relative to memory T cells induced by Ad5 vectors [9], [23]. This suggested that T cells elicited by Ad5 vectors are partially exhausted. To better understand the immunological differences between Ad5 and alternative serotype Ad vaccine vectors, we assessed the expression of multiple co-inhibitory receptors. In this report, we show that Gag-specific CD8 T cells induced by alternative serotype Ad vectors also induced reduced expression of the T cell immunoglobulin and mucin domain (Tim-3) inhibitory receptor relative to Ad5 vectors. Tim-3 is an inhibitory receptor that has been shown to negatively regulate cytokine expression on T cells [15], [24], [25], and is also known to be upregulated alongside PD-1 on functionally exhausted T cells during chronic viral infections such as those with LCMV Cl-13, HCV or HIV. Importantly, CD8 T cells co-expressing both PD-1 and Tim-3 appear to be more functionally exhausted than those expressing either PD-1 or Tim-3 alone [26], [27], [28], [29], [30], [31], [32]. Consistent with these prior reports, low Tim-3 levels in CD8 T cells primed by Ad26 vectors were associated with robust recall CD8 T cell responses following heterologous boosting with modified vaccinia Ankara (MVA) or Ad35. We also demonstrated that lowering the dose of the Ad5 prime led to improved CD8 T cell responses, suggesting a role for antigen burden in dictating the extent of the exhausted T cell response following immunization with Ad5.

## 2 Materials and methods

### 2.1 Mice and infections

Six to 8-week-old female C57BL/6 mice (from Jackson Laboratories) were used for all immunization experiments. Replication-incompetent, E1/E3-deleted Ad5, Ad26, Ad35, and Ad48 vectors expressing SIVmac239 Gag were prepared as previously described [1]. Modified vaccinia Ankara (MVA) expressing SIV Gag-Pol-Env was provided by the U.S. Military HIV Research Program (MHRP). Mice were immunized intramuscularly in both hind leg muscles with Ad vectors at 10^10^ viral particles (vp) per mouse or with escalating doses of Ad5 (10^8^, 10^9^ or 10^10^ vp) per mouse for dose titration experiments. Vector concentration was determined by spectrophotometry against known standards. For boosting experiments, mice were boosted intramuscularly with MVA at ⩽10^7^ plaque-forming units(PFU) per mouse or with Ad35 at 10^9^ vp per mouse. Intramuscular (i.m.) injections were administered in 100 μl phosphate-buffered saline (PBS) injections (50 μl per quadricep). All experiments were performed with approval of the Institutional Animal Care and Use Committee (IACUC).

### 2.2 Intracellular cytokine staining (ICS) assays

Cytokine expression of Gag-specific cellular immune responses in immunized mice were assessed by multiparameter intracellular cytokine staining (ICS) assays. Lymphocytes isolated from the spleen (10^6^) or the liver (10^6^) were incubated for 5 h at 37 °C with 0.2 μg/ml of SIV Gag AL11 peptide (an immunodominant CD8 T cell epitope) together with brefeldin A (GolgiPlug) and monensin (GolgiStop). Cells were stained with anti-mouse CD8-PerCPCy5.5 (53–6.7) and anti-mouse/human CD44 Pacific Blue (IM7) and then were fixed and permeabilized (Cytofix/Cytoperm) prior to intracellular staining with anti-mouse gamma interferon (IFN-γ), anti-mouse tumor necrosis factor alpha (TNF-α), and anti-mouse interleukin 2 (IL-2). All of these reagents were purchased from BD Biosciences.

### 2.3 Surface markers and tetramer binding assays

Single-cell suspensions from blood or homogenized spleen or liver were stained for 30 min at 4 °C with anti-mouse CD8α (53–6.7), anti-mouse/human CD44 (IM7), anti-mouse CD127 (A7R34), anti-mouse CD62L (MEL-14), anti-mouse PD-1 (RMP1-30), anti-mouse Tim-3 (RMT3-23), and Live/Dead Near-IR reagent. All antibodies were purchased from BD Pharmingen, except for CD44 (Biolegend), PD-1 (Biolegend), Tim-3 (Biolegend), and Live/Dead Fixable Near-IR antibody (Invitrogen). H-2D^b^-AL11 biotinylated monomers were obtained from the NIH tetramer facility at Emory University. Fixed cells were acquired using an LSR II flow cytometer (BD Biosciences) and analyzed using FlowJo software (Treestar).

### 2.4 Statistical analysis

Statistical analysis was performed on GraphPad Prism using a two-tailed unpaired Student’s *t* test except for two-tailed paired Student’s *t* test where stated. Data are presented as standard errors of the means (SEM).

## 3 Results

### 3.1 Reduced PD-1 and Tim-3 co-expression on memory CD8 T cells after immunization with alternative serotype adenovirus (Ad) vectors compared to Ad5 vectors

**Fig. 1:**
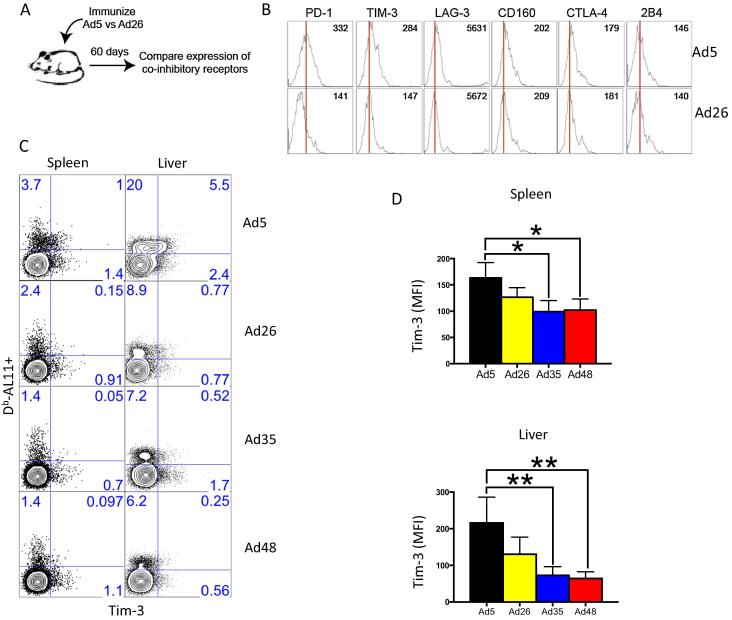
Reduced Tim-3 expression on memory CD8 T cells after immunization with alternative serotype Ad vectors compared to Ad5 vectors. (A) Experiment layout. (B) Representative histograms showing expression of multiple co-inhibitory receptors on Gags-specific (D^b^AL11+) CD8 T cells. (C) Representative FACS plots showing percentage of Gag-specific CD8 T cells in spleen and liver that express Tim-3. (D) Summary of mean fluorescence intensity (MFI) of Tim-3 staining on Gag-specific CD8 T cells in spleen and liver. Tissue data are from approximately day 60 post-immunization. Data in spleen are representative of three independent experiments, with *n* = 4 mice per group per experiment. Data in liver are representative of two independent experiments, with *n* = 4 mice per group per experiment. ^*^p < 0.05; ^**^p < 0.01. Error bars indicate SEM.

To assess the expression of inhibitory receptors on vaccine-elicited CD8 T cells, we immunized C57BL/6 mice intramuscularly with 10^10^ viral particles (VP) of Ad vectors expressing simian immunodeficiency virus (SIV) Gag and evaluated co-inhibitory receptor expression at day 60 (Fig. 1A). Consistent with previous observations [9], [23], PD-1 expression was higher on virus-specific CD8 T cells from Ad5 immunized mice compared to Ad26 immunized mice (Fig. 1B) [9]. Interestingly, we also noticed upregulation of inhibitory Tim-3 following vaccination with Ad5 relative to vaccination with alternative serotype Ad vectors (Fig. 1B and C). Of note, vaccination with the Ad5 vector elicited greater percentages of Gag-specific CD8 T cells expressing Tim-3 compared to vaccination with alternative serotype Ad vectors, and the per-cell expression of Tim-3 was more strikingly different in the liver, with Ad5 inducing two-fold greater levels of Tim-3 expression compared to alternative serotype Ad vectors (p < 0.01) (Fig. 1D). There were no significant differences in the expression of other inhibitory receptors, such as LAG-3, CD160, CTLA-4 and 2B4 (Fig. 1B), suggesting that Ad5 induced a phenotype of partial exhaustion, but not full exhaustion, as is typically observed during chronic viral infection.

**Fig. 2:**
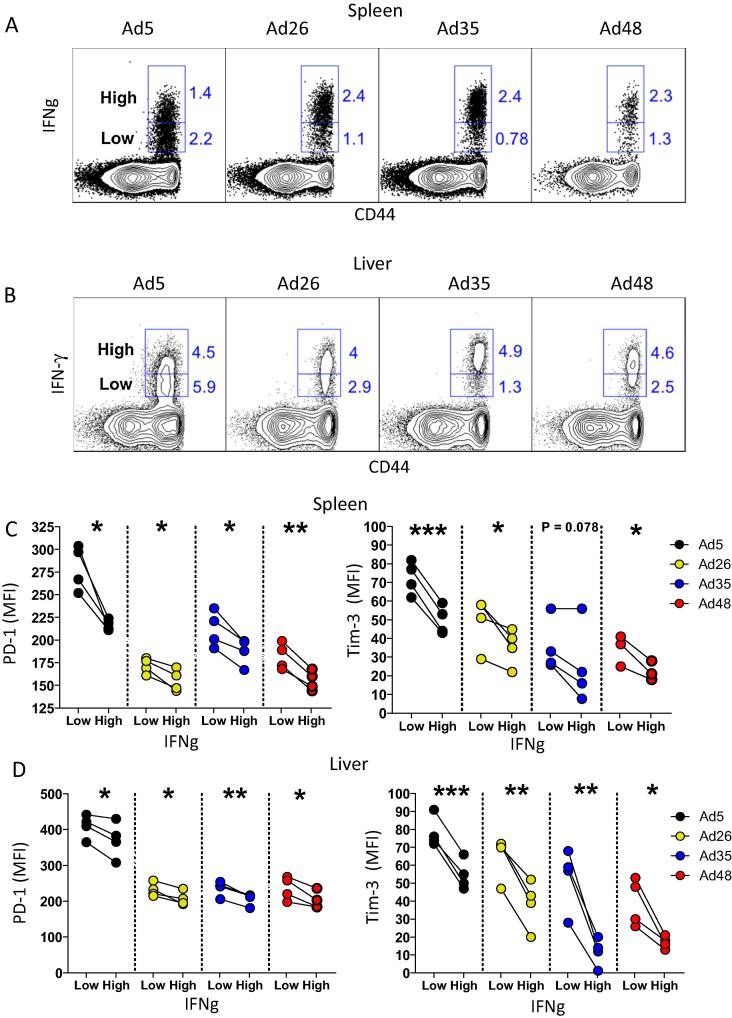
PD-1 and Tim-3 levels on memory CD8 T cells are inversely associated with IFN-γ levels. (A) Representative FACS plots showing Gag-specific specific CD8 T cells in spleen with high or low IFN-γ expression. (B) Representative FACS plots showing Gag-specific specific CD8 T cells in liver with high or low IFN-γ expression. (C) MFI of PD-1 or Tim-3 on Gag-specific CD8 T cells in spleen with high or low IFN-γ expression. (D) MFI of PD-1 or Tim-3 on Gag-specific CD8 T cells in liver with high or low IFN-γ expression. Data are from two experiments, with *n* = 4 mice per group. Statistical analysis was conducted using two-tailed paired Student’s *t* test. ^*^p < 0.05; ^**^p < 0.01; ^***^p < 0.001. Error bars indicate SEM.

Both PD-1 and Tim-3 expression was inversely associated with T cell functionality. Alternative serotype Ad vectors induced greater percentages of IFN-γ^hi^ Gag-specific CD8 T cells as compared to Ad5 (Fig. 2A and B), consistent with our previous results in the LCMV GP system [9]. Moreover, IFN-γ^hi^ CD8 T cells showed lower PD-1 and Tim-3 expression as compared to IFN-γ^low^ CD8 T cells (Fig. 2C and D). Thus, inhibitory PD-1 and Tim-3 expression following Ad vector immunization can be used to assess T cell functionality following vaccination. Although this inverse association between multiple co-inhibitory receptor expression and low cytokine expression has already been established for exhausted CD8 T cells in the context of chronic infection and cancers [15], [25], [33], it has not been evaluated thoroughly on memory CD8 T cells in the context of vaccination. Our data suggest that alternative serotype Ad vectors induce highly functional CD8 T cell responses with low expression of co-inhibitory PD-1 and Tim-3 receptors and high cytokine production in response to antigen.

### 3.2 Long-term enhancement in memory CD8 T cell conversion and improved functionality after vaccination with Ad26 vector compared to Ad5 vector

**Fig. 3:**
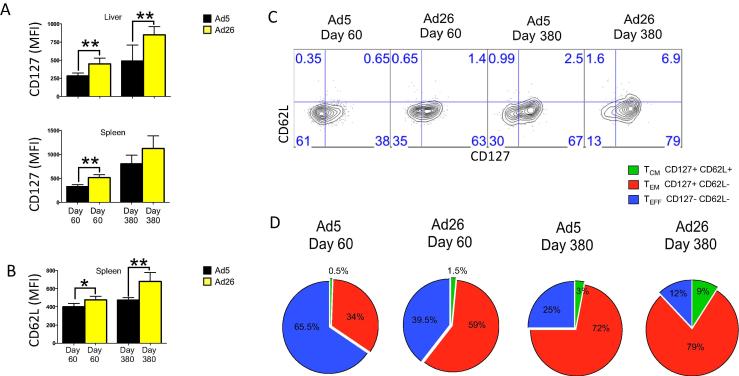
Differences in central memory CD8 T cell differentiation are also observed at late time points following immunization with Ad5 or alternative serotype Ad vectors. (A) MFI of memory marker CD127 expresion on Gag-specific CD8 T cells in the liver and spleen. (B) MFI of lymphoid trafficking marker CD62L expression on Gag-specific CD8 T cells in the spleen. (C) Representative FACS plots showing expression of CD127 and CD62L on Gag-specific CD8 T cells in the spleen. (D) Pie charts represent the proportion of Gag-specific CD8 T cells in spleen that are of effector (T_EFF_), effector memory (T_EM_), or central memory (T_CM_) phenotypes (*n* = 4). Day 60 Ad5 versus Ad26; T_EFF_ P = 0.0028; T_EM_ P = 0.0036; T_CM_ P = 0.0002. Tissue data are from approximately day 60 or day 380 post-immunization as indicated. Data are representative of two independent experiments, with *n* = 4 mice per group per experiment. ^*^p < 0.05; ^**^p < 0.01. Error bars indicate SEM.

We previously showed that following vaccination with Ad5, there is a slower effector memory to central memory CD8 T cell differentiation relative to vaccination with alternative serotype Ad vectors (CD8 T cells elicited by Ad5 exhibit a CD127^lo^ CD62L^lo^ phenotype at day 60 post-vaccination) [9]. However, memory T cell differentiation is a continuous process following vaccination or acutely controlled infection [34], [35], [36], [37], [38], and we reasoned that it is possible that central memory conversion with cytokine upregulation may occur at a later time point following Ad5 vaccination. We thus interrogated the long-term immune phenotypes following Ad5 or Ad26 vaccination to specifically assess whether CD8 T cells elicited by Ad5 can eventually differentiate into highly functional central memory subsets. Mice were sacrificed at early memory time points (day 60) or late memory time points (day 380) after vaccination, and the phenotype and functionality of CD8 T cell responses was examined. At all time points, Ad26 showed improved expression of the memory markers CD127 and CD62L relative to Ad5, and there was a greater fraction of CD8 T cells that had differentiated into central memory (CD127+ CD62L+) subsets (mean percentage for day 380 for Ad5 was 3.3% and for Ad26 was 9.6%) (Fig. 3A–D).

**Fig. 4:**
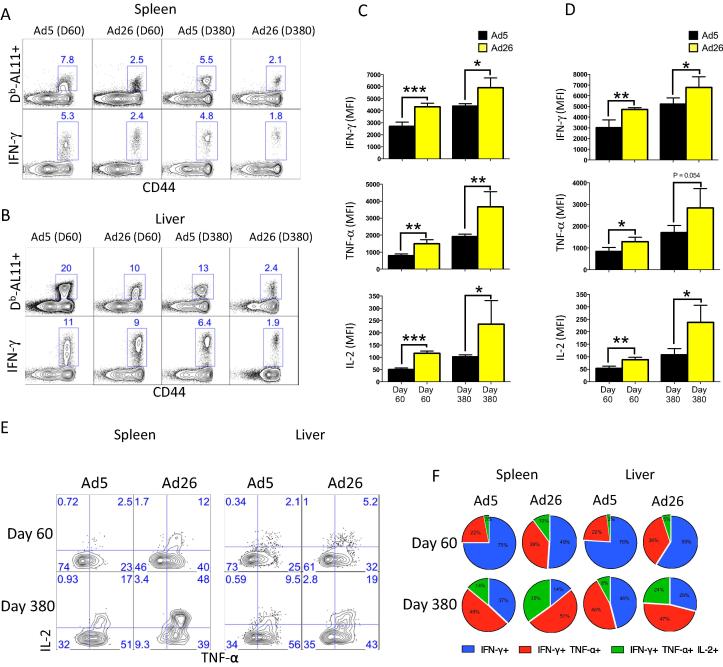
Immunization with Ad26 results in increased functional CD8 T cell responses compared to Ad5. (A) Representative FACS plots showing tetramer to cytokine ratio in Gag-specific CD8 T cells from the spleen. (B) Representative FACS plots showing tetramer to cytokine ratio in Gag-specific CD8 T cells from the liver. (C) MFI of IFN-γ, TNF-a, and IL-2 expression in Gag-specific CD8 T cells in spleen after peptide stimulation. (D) MFI of IFN-γ, TNF-a, and IL-2 expression in Gag-specific CD8 T cells in liver after peptide stimulation. (E) Representative FACS plots in spleen showing percentages of Gag-specific CD8 T cells co-expressing TNF-a and IL-2 in spleen and liver. Samples were gated on IFN-γ^+^ Gag-specific CD8 T cells. (F) Summary showing the proportion of functional CD8 T cells in spleen and liver coproducing IFN-γ, TNF-a, and IL-2 upon stimulation with peptide. Tissue data are from day 60 or day 380 post-immunization. Data are representative of two independent experiments, with n = 4 mice per group per experiment. ^*^p < 0.05; ^**^p < 0.01; ^***^p < 0.001. Error bars indicate SEM.

Although Ad5 induced higher magnitudes of Gag-specific CD8 T cells especially in the liver (relative to Ad26), these cells appeared to be partially exhausted, evidenced by a low tetramer to cytokine ratio (Fig. 4A and B) and decreased per cell expression of IFN-γ, TNF-α, and IL-2 even after day 380 post-immunization (Fig. 4C and D). In addition, the frequencies of triple producing (IFN-γ, TNF-α, and IL-2) CD8 T cells were greater in Ad26 vaccinated mice relative to Ad5 vaccinated mice at both early and late time points (Fig. 4E and F). Taken together, the enhanced central memory CD8 T cell conversion and improved CD8 T cell functionality after immunization with Ad26 compared to Ad5 were still observed at later stages of the immune response. Moreover, these data demonstrated permanent induction of a partially exhausted, effector-like CD8 T cell response by Ad5.

### 3.3 Robust anamnestic CD8 T cell responses after vaccination with Ad26 vector

**Fig. 5:**
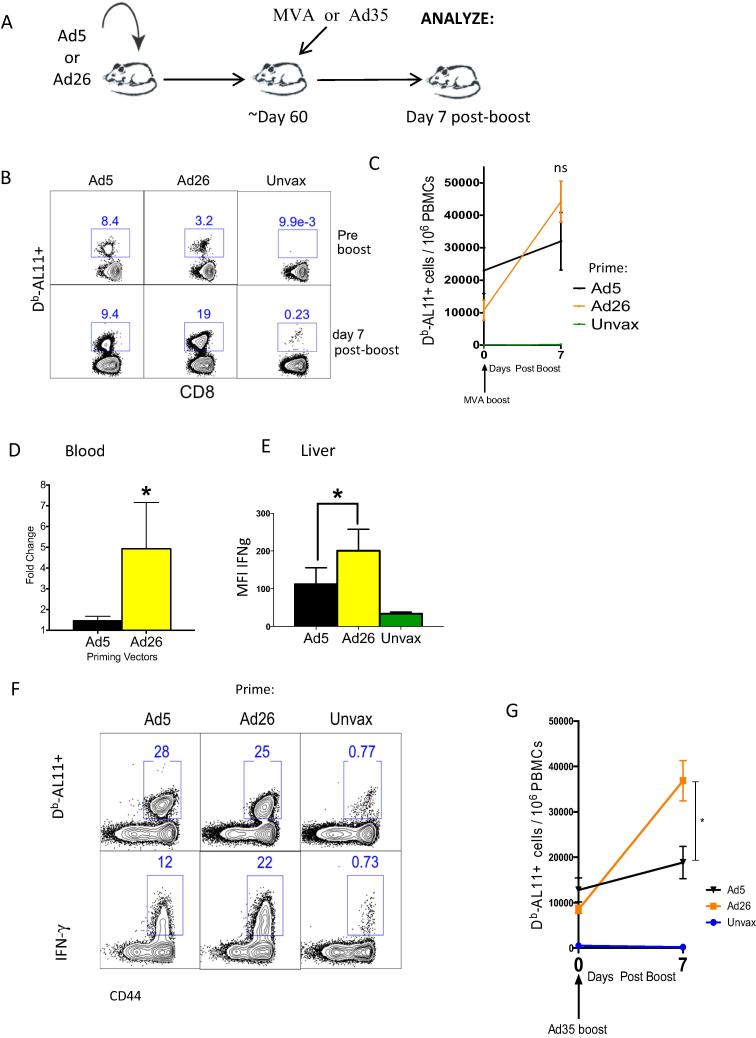
Priming with Ad26 vector results in improved recall responses after MVA or Ad35 boosting compared to priming with Ad5 vector. (A) Experimental outline. (B) Representative FACS plots showing the percentage of Gag-specific CD8 T cells before and after MVA boost in blood. (C) Summary showing the number of Gag-specific CD8 T cells before and after MVA boost in blood. (D) Summary showing the fold-increase of Gag-specific CD8 T cells in blood after MVA boost. (F) MFI of IFN-γ expression by Gag-specific CD8 T cells from liver. (F) Representative FACS plots showing functionality of Gag-specific CD8 T cells in liver as measured by tetramer and cytokine expression. (G) Summary showing the number of Gag-specific CD8 T cells before and after Ad35 boost in blood. Unprimed mice vaccinated with MVA or Ad35 were used as controls (Unvax). Data are representative of two independent experiments, with n = 4 mice per group per experiment. ^*^p < 0.05; ^**^p < 0.01.

We next used an MVA boost regimen to compare anamnestic CD8 T cell responses in mice primed with Ad5 or Ad26 expressing SIV Gag (see Materials and Methods). Mice were sacrificed at day 7 post MVA boost to compare the peak anamnestic expansion of Gag-specific CD8 T cells in both blood and tissues (Fig. 5A). Note that an Ad5 prime induced greater levels of Gag-specific CD8 T cells relative to Ad26 as previously shown [9], [39]. However, following an MVA boost, Ad26-primed CD8 T cells showed a robust recall expansion (difference between pre and post-boost for Ad5 was 1.47-fold, p = 0.0318; for Ad26 was 4.93-fold, p = 0.0031; Ad26 recall expansion was 3.4-fold greater compared to Ad5, p < 0.05) (Fig. 5B–D). Following Ad26/MVA prime-boost vaccination, there was also a greater per cell IFN-γ expression in Gag-specific CD8 T cells in the liver (Fig. 5E) (P = 0.05) with increased tetramer to cytokine ratio (Fig. 5F) as compared to Ad5/MVA prime-boost vaccination. A similar trend was observed with an Ad35 boost suggesting the generalizability of these observations (Fig. 5G). The improved recall expansion of Ad26 primed CD8 T cells over those primed by Ad5 appeared irrespective of the boosting vector.

### 3.4 Improved anamnestic CD8 T cell responses after vaccination with low dose Ad5 vector

**Fig. 6:**
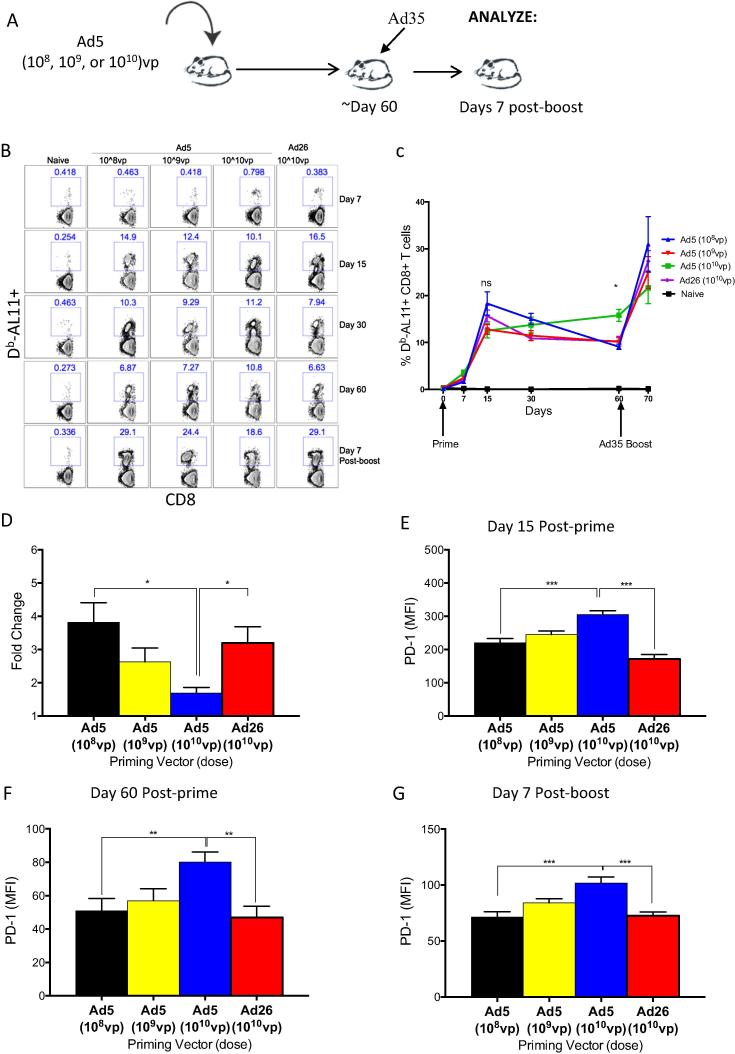
Lowering the priming dose of Ad5 vector partially improves the phenotype and the recall responses after Ad35 boosting. (A) Experimental outline. (B) Representative FACS plots showing the percentage of Gag-specific CD8 T cells on indicated days following Ad5 or Ad26 prime and 7 days after Ad35 boost in blood. (C) Summary showing the kinetics of Gag-specific CD8 T cells before and after Ad35 boost in blood. (D) Summary showing the fold-increase of Gag-specific CD8 T cells in blood after Ad35 boost. (E–G) MFI of PD-1 on Gag-specific CD8 T cells in blood at (E) peak time response (Day 15) and at (F) memory (Day 60) following priming with indicated doses of Ad5, and on (G) day 7 after Ad35 boost. Data are representative of two independent experiments, with n = 5 mice per group per experiment. ^*^p < 0.05; ^**^p < 0.01; ^***^p < 0.001.

A previous study showed that lowering the dose of an Ad5 prime can improve vaccine-elicited CD8 T cells responses [40]. We therefore reasoned that the impaired recall immune response seen with Ad5 primed mice (which included higher inhibitory receptor expression and lower cytokine expression relative to Ad26) may be overcome by reducing the priming dose in a heterologous prime-boost vaccine regimen. We compared the CD8 T cell recall response in mice primed with escalating doses of Ad5 (10^8^, 10^9^ or 10^10^ vp), and boosted at day 60 post prime with Ad35 or MVA. Mice were bled longitudinally to assess the magnitude and phenotype of antigen specific CD8 T cells in blood (Fig. 6A). All doses of Ad5 elicited similar peak CD8 T cell responses at day 15 post-prime, but the low dose Ad5 prime (10^8^ and 10^9^ vp) were associated with more pronounced CD8 T cell contraction compared to high dose Ad5 prime (10^10^ vp) (Fig. 6B and C) (p < 0.001). However, the fold recall expansion of memory CD8 T cell was significantly greater with low dose compared to high dose Ad5 prime by day 7 post-boost (fold-increase for low dose was 2.39-fold, p < 0.0001; fold-increase for high dose was 0.99-fold, p = 1) (Fig. 6B–D).

Finally, we assessed the expression of PD-1 on antigen-specific CD8 T cells and observed that antigen-specific CD8 T cells in mice primed with low dose Ad5 expressed significantly lower PD-1 relative to high dose Ad5-primed mice at all time points, including at the peak of the primary response (p < 0.0001), during the memory response (p = 0.0059), and after Ad35 boost (p < 0.0005) (Fig. 6E–G). A similar trend was observed following MVA boost (data not shown). Therefore, our findings confirm and extend previous reports [40] and further suggest that lowering the priming dose of Ad5 100-fold can mitigate the exhausted phenotype and improve memory recall following Ad5 vaccination, resulting in T cell responses that are similar to those of Ad26.

## 4 Discussion

Substantial biological differences between Ad5 and alternative serotype Ad vectors, including differences in tropism [22], [23], [24], primary receptor usage [19], [22], [24], [25], [26], [27], and triggering of innate immune responses [28], [29], [30], [31], [32], [33], may help to explain their observed immunologic differences. Our laboratory and others have previously reported that although Ad5 is highly immunogenic, it induced CD8 T cells with a more terminally differentiated phenotype, partial exhausted phenotype, and defective recall capability [9], [23], [41]. Our prior observations were reported using recombinant Ad vectors expressing lymphocytic choriomeningitis virus glycoprotein antigens (LCMV GP). Here, we have generalized these observations to the SIV Gag system with two different boosting platforms. Moreover, we show that in addition to PD-1 upregulation, CD8 T cells elicited by Ad5 vectors exhibit Tim-3 upregulation. These immunological differences may help to explain the distinct recall potential of CD8 T cells elicited by Ad5 and alternative serotype Ad vectors when delivered at high doses.

The mechanism for the immune dysfunction induced by Ad5 is not well understood, and the role of antigen persistence, dendritic cell priming, and various innate immune pathways remain to be determined. Our prior data show that cellular tropism does not appear to account for the immune defects that are observed in Ad5-induced CD8 T cells, as hexon hypervariable region-modified Ad5 (Ad5HVR48), which exhibits a tropism similar to that of Ad48, induces T cell immune dysfunction similar to Ad5 [42]. We have also observed a similar pattern of memory CD8 T cell differentiation following immunization of rhesus macaques with Ad5 versus Ad26 (data not shown).

PD-1+ Tim-3+ T cells are also known to be more functionally impaired than CD8 T cell populations that remain single-positive for either marker, as suggested by prior studies [26], [27], [28], [29], [30], [31], [32]. Lower levels of the co-inhibitory receptors PD-1 and Tim-3, and increased levels of memory markers on CD8 T cells elicited by Ad26 vaccination may help to explain the enhanced anamnestic potential relative to Ad5 vaccination. It has also been suggested that lowering the dose of Ad5 vaccines may improve the functionality of CD8 T cells [40]. Our study confirms and extends this prior report by showing improved fold expansion of CD8 T cells and low inhibitory receptor expression in low dose Ad5 vaccine groups relative to high dose Ad5 vaccine groups. Since Ad26 is less immunogenic at low doses (10^7^ VP induce negligible responses in mice) [23], our data cannot compare CD8 T cell function with high versus low doses of Ad5 and Ad26. We therefore assessed Ad5-induced CD8 T cell responses at both high and low doses to demonstrate that CD8 T cells are more functional with low doses of this vector.

In conclusion, we expand previous comparative findings on Ad5 and alternative serotype Ad vectors by providing additional data showing that the phenotype, functionality, and recall potential of memory CD8 T cells elicited by alternative serotype Ad and Ad5 vectors are substantially different in their phenotype and long-term recall capacity. Importantly, we confirm that these observations are reproducible in multiple prime boost regimens. These findings advance our current understanding of Ad vectors for development as vaccine candidates against HIV-1.
